# Orthopedic healthcare in the time of COVID-19: Experience of the orthopedic surgery department at Mustapha Bacha Hospital, Algeria

**DOI:** 10.1016/j.amsu.2020.05.025

**Published:** 2020-05-23

**Authors:** Nadhir Meraghni, Riad Benkaidali, Mohamed Derradji, Zoubir kara

**Affiliations:** Department of Orthopedic Surgery. Centre Hospitalier Universitaire Mustapha Bacha, Algiers, Algeria

**Keywords:** COVID-19, Pandemic, Orthopedic surgery

## Abstract

In response to the global health emergency, which has been raised to its highest level as a consequence of the coronavirus disease 2019 (COVID-19), urgent and aggressive actions were taken by health institutions across the world to stop the spread of the disease while ensuring continuity of vital care.

This article outlines the urgent measures put in place by the orthopedic surgery department at Mustapha Bacha Hospital in response to the COVID-19 pandemic.

The COVID-19 pandemic is one of the largest global healthcare crises in nearly a century. The coronavirus disease started in Wuhan, China in late 2019 and then spread rapidly worldwide [1]. On March 11, 2020, the World Health Organization (WHO) characterized Covid-19 as a pandemic [2]. The first confirmed case of COVID-19 in Algeria was reported on February 25, 2020. At the time of writing this paper, Algeria is ranked first in Africa in term of registered COVID-19 deaths.

The COVID-19 pandemic put immense pressure on healthcare systems across the world, including orthopedic practice. Although, the daily activity of orthopedic surgery has been markedly affected, surgical teams have responded effectively to the COVOD-19 pandemic while maintaining the continuity of health care and ensuring protection of medical staff and patients [3].

At first glance, orthopedic surgeons are not considered frontline healthcare staff in the fight against COVID-19. However, as part of the larger healthcare system, they have an important role to play in reining in this pandemic [1,4].

The Mustapha Bacha Hospital is the biggest and major university hospital in Algeria. As an important part of the hospital, orthopedic surgery department has quickly adapted its program to face the unprecedented challenges due to the COVID-19 pandemic [5].

Orthopedic department is a multi-specialty care including spine, upper limbs, lower limbs and high volumes of emergency department visits and trauma cases. Additionally, it is a bone tumour surgery reference center. The orthopedic surgical activity at Mustapha Bacha Hospital has been severely disrupted during this pandemic [3].

We benefit from the experience of our colleagues around the world to help us adopt the most effective strategy to face this challenging period [6].

This article outlines the experience and response of the orthopedic surgery department at Mustapha Bacha Hospital during the COVID-19 pandemic [5].

## 1 Inpatient surgical management

Elective, non-urgent surgical cases have been postponed or cancelled [4]. This decision made by the head of department with concertation with all the senior surgeons was based on both medical and logistical considerations. It allows to free-up beds and increase the hospital capacity to treat suspected or COVID-19 positive patients requiring hospitalization [4]. It permits the reduction of health workers while maintaining urgent surgery capabilities [3].

Postponing surgeries help to minimize spread of infection between symptomatic and asymptomatic patients and health care staff [6]. When all elective surgical procedures have been cancelled, only trauma and tumour cases were permitted to proceed [4]. To date, we have been able to perform all trauma and urgent tumour surgeries without any substantial delays [5]. We have also continued to accept and treat patients transferred from health units across the country, in particular tumour and complex trauma cases to provide vital orthopedic care in a timely manner [5].

## 2 Operating room

Given the increased risk of coronavirus transmission in hospitals in general and operating theaters in particular, special personal protective measures must be provided. Because of the low number of COVID-19 test kits available at the hospital and which are reserved for patients with high suspicion of COVID-19 infection, each patient admitted to the orthopedic department and the operating room is considered like COVID-19 positive even if they are otherwise healthy adults with no comorbidities. Surgeons must ensure utmost care is provided to patients in the preoperative, intraoperative, and postoperative settings to minimize risks of infection [4,7,8]. Personnel entering operating theatre must be reduced [9]. All traffic in and out of the operating theater should be minimized [6]. Intraoperatively, full personal protection equipment including N95 FFP2 masks, protective glasses and face shields should be used even if it reduces the clarity of verbal communication between theatre staff [10]. Consideration should be given to surgical approaches that could decrease operating staff exposure and shorten case duration [6]. Surgical team should be kept to the minimum, whenever possible [3,4,11]. In our department, we deplore the absence of N95 masks supplied to our staff. We only benefit from simple surgical masks to operate on patients during this pandemic. Surgical face masks are not designed for personnel protection and do not closely fit around the face and mouth. Their design is intended for preventing contamination of the surgical wound from the aerosols generating by the surgical team [9]. We have received face shields made and supplied by generous donors.

## 3 Outpatient clinic management

Although a certain number of post-operative in person patient visits still occur or when urgent issues arise, actions were taken to maintain a reasonable workload and minimize staff and patient contacts [5,12]. At the clinic, social distancing is practiced at all times [12].

Physicians have also been advised to prolong the duration between non-urgent follow-ups to reduce patient overcrowding in hospitals [3,4,11].

The emergence of such a crisis provides a timely opportunity for us to reflect and evaluate the use of novel technologies in the workplace. This includes the adoption of telemedicine initiatives, allowing patients to be consulted and followed-up in the comfort of their own homes [4,11,[13], [14], [15]]. Where possible, consultations are done through the telephone and this helps to allay patients’ anxiety [3]. Many countries over the world are now conducting telephone outpatient clinics in light of COVID-19 [9].

## 4 General measures

Although non-urgent surgical procedures have been postponed until the current situation improves, orthopedic surgeons must ensure that appropriate quality of care provided to patients is maintained. Physical and social distancing must be respected between health workers and patients. With the increased number of COVID-19 cases in the hospital, the intensive care and pulmonology units were quickly saturated. To align with other hospital departments, a COVID19 unit has been set up in the orthopedic surgery department to receive COVID-19 positive patients. There is a single entrance in this unit and dedicated nursing staff to avoid contact with the rest of patients admitted for orthopedic conditions.

The COVID-19 crisis has resulted in people working outside their specialty [1]. The orthopedic community has banded with their medical and surgical colleagues in the battle against COVID-19 [4]. During this COVID-19 crisis, orthopedic surgeons and residents, together with their counterparts from other specialties, have been requisitioned for shifts in the special COVID-19 ward at the emergency department to assist with the screening of suspected cases [4]. The COVID-19 shifts were planned to involve all the medical and surgical departments of the Mustapha Bacha hospital. Our team was composed of 2 senior surgeons and 2 junior surgeons (residents) with a rotation system to involve all the surgeons and residents of the orthopedic surgery department.

Any plans need to be developed with a recognition that the severity of the situation and the availability of resources may change on a daily basis [6]. Everyone should remain flexible, adaptable and ready to pivot rapidly to changing events [16].

## 5 Medical education and orthopedic training

During this crisis, all teaching conferences for residents have been suspended. In addition, elective surgical procedures have been cancelled [4]. As a consequence, residents training have been significantly impacted.

To overcome this situation, thanks to the contribution of technology, we have offered our residents teaching programs using the ZOOM videoconference platform which allows the medical staff to stay in touch while staying safe at home.

## 6 Staff wellbeing

Fearing COVID-19 spread to their families, many health workers have taken the decision to self-isolate. Also, there is a widespread concern causing stress and anxiety [10], which resulted in a negative impact on the mental health of health workers.

It is crucial that colleagues support each other and recognize that everyone is working outside of their zone of comfort [9].

## 7 Conclusion

We believe that sharing experience between health care actors allows us to have an effective strategy to provide the very best care to our patients during the COVID-19 pandemic. We hope, once getting out of this crisis, we will continue to work together as valued team members.

## Funding

The authors received no external funding for this research.

## Provenance and peer review

Not commissioned, Editor reviewed.

## Ethical approval

N/A.

## Author contribution

Nadhir Meraghni=concept and design, writing the paper, validation.

Riad Benkaidali=concept and design, validation.

Mohamed Derradji=validation, supervision.

Zoubir Kara=validation, supervision.

## Registration of research studies

1. Name of the registry:

2. Unique Identifying number or registration ID:

3. Hyperlink to your specific registration (must be publicly accessible and will be checked):

## Guarantor

Nadhir Meraghni.

## Declaration of competing interest

On behalf of all authors, the corresponding author states that there is no conflict of interest.

## References

[1] Ashford R.U., Nichols J.S., Mangwani J. (2020 Apr). Annotation: the COVID-19 pandemic and clinical orthopaedic and trauma surgery. J Clin Orthop Trauma.

[2] Shi Y., Wang J., Yang Y., Wang Z., Wang G., Hashimoto K. (2020 Apr 1). Knowledge and attitudes of medical staff in Chinese psychiatric hospitals regarding COVID-19. Brain Behav Immun - Health.

[3] Ahmed S., Leong Glenn T.W., Chong Y.-L. (2020 Apr). Surgical response to COVID-19 pandemic: a Singapore perspective. J. Am. Coll. Surg..

[4] Chang Liang Z., Wang W., Murphy D., Po Hui J.H. (2020 Apr 20). Novel coronavirus and orthopaedic surgery: early experiences from Singapore. JBJS [Internet]. https://journals.lww.com/jbjsjournal/Citation/9000/Novel_Coronavirus_and_Orthopaedic_Surgery__Early.99807.aspx.

[5] Schwarzkopf R., Maher N.A., Slover J.D., Strauss E.J., Bosco J.A., Zuckerman J.D. (2020). The response of an orthopedic department and specialty hospital at the epicenter of a pandemic: the NYU langone health experience. J. Arthroplasty.

[6] Brindle, Mary MD, MPH*; gawande, atul MD, MPH† managing COVID-19 in surgical systems, Ann. Surg.: Mar 23, 2020 - Volume Publish Ahead of Print - Issue - doi: 10.1097/SLA.0000000000003923. 10.1097/SLA.0000000000003923 PMC7188040 32209891

[7] Awad M.E., Rumley J.C.L., Vazquez J.A., Devine J.G. (2020 Apr 15). Peri-operative considerations in urgent surgical care of suspected and confirmed COVID-19 orthopedic patients: operating rooms protocols and recommendations in the current COVID-19 pandemic. JAAOS - J Am Acad Orthop Surg.

[8] Tan Z., Phoon P.H.Y., Zeng L.A., Fu J., Lim X.T., Tan T.E. (2020 Mar). Response and operating room preparation for the COVID-19 outbreak: a perspective from the national heart centre in Singapore. J cardiothorac vasc anesth. 2020 mar;S1053077020303001.4. Viswanath A, monga P. Working through the COVID-19 outbreak: rapid review and recommendations for MSK and allied heath personnel. J Clin Orthop Trauma.

[9] Viswanath A., Monga P. (2020 Mar). Working through the COVID-19 outbreak: rapid review and recommendations for MSK and allied heath personnel. J Clin Orthop Trauma.

[10] Ellis R. (2020). Operating during the COVID-19 pandemic: how to reduce medical error. Br. J. Oral Maxillofac. Surg..

[11] Giacomo P., Damiano S., Elena D., Giulia B., Vincenzo S. (2020 Apr). CoViD-19 and Ortho and Trauma Surgery: the Italian Experience.

[12] Tay K., Kamarul T., Lok W.Y., Mansor M., Li X., Wong J., Saw A. (2020). COVID-19 in Singapore and Malaysia: rising to the challenges of orthopaedic practice in an evolving pandemic. Malaysian Orthopaedic Journal.

[13] Vaccaro A.R., Getz C.L., Cohen B.E., Cole B.J., Donnally C.J.I. (2020 Apr 15). Practice management during the COVID-19 pandemic. JAAOS - J Am Acad Orthop Surg.

[14] Loeb A.E., Rao S.S., Ficke J.R., Morris C.D., Riley L.H.I., Levin A.S. (2020 Apr 15). Departmental experience and lessons learned with accelerated introduction of telemedicine during the COVID-19 crisis. JAAOS - J Am Acad Orthop Surg.

[15] Parisien R.L., Shin M., Constant M., Saltzman B.M., Li X., Levine W.N. (2020 Apr 15). Telehealth utilization in response to the novel coronavirus (COVID-19) pandemic in orthopaedic surgery. JAAOS - J Am Acad Orthop Surg.

[16] Massey P.A., McClary K., Zhang A.S., Savoie F.H., Barton R.S. (2020 Apr 15). Orthopaedic surgical selection and inpatient paradigms during the coronavirus COVID-19 pandemic. JAAOS - J Am Acad Orthop Surg.

